# Role of Extracellular Vesicles in Chronic Post-Embolic Pulmonary Hypertension: Data from an Experimental Animal Model and Patients

**DOI:** 10.3390/biomedicines13061499

**Published:** 2025-06-18

**Authors:** Elva Mendoza-Zambrano, Verónica Sánchez-López, Belén Gómez-Rodríguez, Inés García-Lunar, Daniel Pereda-Arnau, Luis Jara-Palomares, Teresa Elías-Hernández, Ana García-Álvarez, Remedios Otero-Candelera

**Affiliations:** 1 Medical Surgical Unit of Respiratory Diseases, Virgen del Rocio University Hospital, 41013 Seville, Spain; 2 Institute of Biomedicine of Seville (IBiS) Virgen del Rocio University Hospital/CSIC/University of Seville, 41013 Seville, Spain; 3 Department of Social and Health Sciences, San Isidro Center, 41092 Seville, Spain; 4 National Center of Cardiovascular Research (CNIC), Cardiology Department, La Moraleja University Hospital, 28050 Madrid, Spain; 5 Biomedical Research Center for Cardiovascular Diseases (CIBERCV), 28029 Madrid, Spain; 6 Hospital Clinic of Barcelona, Institute of Biomedical Research August Pi i Sunyer (IDIBAPS), University of Barcelona, 08007 Barcelona, Spain; 7 Biomedical Research Center Network for Respiratory Diseases (CIBERES), Carlos III Health Institute, 28029 Madrid, Spain

**Keywords:** pulmonary embolism, chronic thromboembolic pulmonary hypertension, extracellular vesicles, endothelial dysfunction, vascular remodeling

## Abstract

**Background**: The pathogenesis of chronic thromboembolic pulmonary hypertension (CTEPH) involves a multifaceted interplay of factors, including incomplete thrombus resolution, endothelial dysfunction, and vascular remodeling. Recent studies have highlighted the role of extracellular vesicles (EVs) in vascular diseases, suggesting their potential involvement in CTEPH progression. This study aims to investigate the role of EVs from various cellular sources in the development of CTEPH. **Methods**: An experimental study was conducted using 11 male three-month-old Large-White pigs. The EVs of endothelial origin (EEVs; CD146+), leukocyte-derived EVs (LEVs; CD45+, CD44+), and consistent with mesenchymal-origin EVs (CD90+, CD105+) were quantified. Measurements were taken at baseline, after the first embolization, and prior to each subsequent weekly embolization. Embolizations were repeated until chronic pulmonary hypertension (PH) was generated. Based on these findings, a clinical case-control study was performed involving nine patients previously diagnosed with CTEPH and 18 patients with pulmonary embolism who did not develop CTEPH after two years of follow-up. **Results**: The experimental study, consistent with the mesenchymal-origin EVs, exhibited a progressive decrease below baseline levels; LEVs decreased after PH was established, while EEVs remained elevated throughout the study. Subsequently, in the clinical case-control study, CD45+ LEVs emerged as a significant association of CTEPH, with an odds ratio (OR) of 21.25 (95% CI: 1.91–236.00; *p* = 0.013). **Conclusions**: Inflammation involving LEVs and EEVs plays a crucial role in sustaining the vascular alterations leading to pulmonary vasculature remodeling in CTEPH.

## 1. Introduction

Chronic thromboembolic pulmonary hypertension (CTEPH) is a rare and potentially life-threatening condition characterized by the persistence of thrombotic material within the pulmonary arterial vasculature, despite adequate anticoagulant therapy following a pulmonary embolism (PE) event. The pathogenesis of CTEPH involves a complex interplay of factors, including incomplete resolution and organization of the initial thrombotic material, leading to the formation of obstructive fibrous plugs that impair pulmonary blood flow and trigger vascular remodeling and defects in angiogenesis [1,2,3].

After a PE event, venous thrombi undergo a structural transformation, shifting from being primarily composed of erythrocytes to being predominantly formed by fibrin and collagen, which entails the recruitment of leukocytes. Neutrophils are the first to be recruited, attracted by interleukin-8, followed by monocyte-derived macrophages, which are the most important effectors in the thrombus dissolution. These macrophages are attracted by chemotactic protein-1 (CCL2) with the objective of restoring blood flow or promoting neovascularization. Endothelial cells also accelerate thrombus resolution, promoted by vascular endothelial growth factor (VEGF) [4,5,6]. However, the precise pathogenesis and mechanisms that generate CTEPH remain unclear [7,8]. The evidence of endothelial dysfunction is mainly based on studies of Pulmonary Arterial Hypertension (PAH) [1,9,10,11] and remains limited in the context of CTEPH.

Extracellular vesicles (EVs) are particles naturally released from the cell that are delimited by a lipid bilayer and cannot replicate. EVs are released from various cell types as part of regular homeostasis and intercellular communication [12,13]. They also contain DNA, RNA, microRNA (miRNA), and other small non-coding RNAs. Notably, their RNA molecules are increasingly recognized as messengers of the cell [14]. In recent years, it has been studied how circulating EVs seem to be related to endothelial dysfunction. The vascular endothelium is a dynamic and versatile tissue that serves as the interface between the bloodstream and the surrounding tissues, playing a crucial role in the maintenance of vascular homeostasis. This delicate balance is maintained through complex and interconnected signaling networks, including the phosphatidylinositol 3-kinase (PI3K)/Akt pathway, the Fas/FasL apoptotic pathway, and the nuclear factor-κB (NF-κB) inflammatory pathway [15,16,17]. Specifically, the PI3K/Akt signaling cascade is a central regulator of endothelial function, governing critical processes such as the modulation of endothelial nitric oxide synthase activity, which, in turn, influences angiogenesis and vascular tone [18]. In parallel, the Fas/FasL system mediates endothelial apoptosis, with the engagement of Fas by its ligand FasL triggering a cascade of caspase activation that ultimately leads to programmed cell death [19]. Furthermore, the NF-κB pathway orchestrates the inflammatory response in endothelial cells, promoting the release of proinflammatory cytokines such as interleukin-1β through the formation of complexes with nucleotide-binding oligomerization domain-like receptor protein 3 (NLRP3) and pro-caspase-1 [20]. These three processes play a crucial role in the activation of endothelial extracellular vesicles [21].

The leukocyte–endothelial interaction during the inflammatory process is well established and occurs through the interaction of CD44+ present on the surface of some leukocytes with glycosaminoglycan hyaluronate, a main component of the extracellular matrix [22]. Mesenchymal stem cells (MSCs) secrete substances through EVs that regulate physiological processes and the expression of target genes [23]. They can suppress the activation of signal transducer and activator of transcription (STAT) 3 through the transfer of miRNA, thereby inhibiting hyperproliferation pathways in pulmonary hypertension [24,25,26]. On the other hand, CD146+ expressed in arterial endothelial cells plays an important role during vascular repair, thus having a causal effect during vascular remodeling [27]. Understanding how different types of extracellular vesicles may serve as disease biomarkers, due to their ability to transmit information between cells or trigger signaling pathways, could aid in developing new diagnostic and therapeutic methods.

The aim of our study was to investigate the role of EVs from different cellular sources in the development of CTEPH. We conducted an experimental study in an animal model to identify relevant EVs that could serve as biomarkers throughout the CTEPH generation process. Then, in a case-control study of CTEPH patients, we investigated the presence of the same cell-derived EVs.

## 2. Materials and Methods

### 2.1. Experimental Study

*Subjects*: The study included 11 male 3-month-old healthy Large-White pigs.

*Procedures*: Nine pigs (intervention group) underwent weekly pulmonary embolizations using 300-micron polydextran microspheres. Hemodynamic data were collected via right heart catheterization (RHC) using a Swan–Ganz catheter inserted through the femoral vein. Measurements were taken at baseline (B), after the first embolization (FE), as well as prior to each subsequent weekly embolization. The first embolization was performed one week after the baseline hemodynamic measurement. Embolizations were repeated once a week until chronic PH was generated, defined as mean pulmonary arterial pressure (PAP) ≥ 25 mmHg at rest. Final hemodynamic reassessment (F) was performed six weeks later to confirm the generation of chronic stable pulmonary hypertension (PH). Blood extraction (10 mL) for EVs analysis was conducted at baseline, after FE, upon reaching pulmonary hypertension (PAP ≥ 25 mmHg), and at the final time point. Two pigs served as a control group, receiving no interventions. These animals maintained stable pulmonary artery pressures (PAP) throughout the follow-up period (mean PAP: 15.2 ± 1.8 mmHg at baseline vs. 15.8 ± 2.1 mmHg at study end). The study protocol was approved by the Institutional Animal Research Committee (PA 1411) of the Centro Nacional de Investigaciones Cardiovasculares Carlos III (CNIC), where the experimental study was developed.

### 2.2. Clinical Case-Control Study

A case-control study was conducted to investigate the association between EVs and the diagnosis of CTEPH. Cases included patients diagnosed with CTEPH, while controls were patients who had experienced pulmonary embolism (PE) but did not develop CTEPH after two years of follow-up.

#### 2.2.1. Participants

*Cases:* Nine patients with confirmed CTEPH were selected. Inclusion criteria encompassed a clinical and hemodynamic diagnosis of CTEPH, aged between 18 and 80 years, and provision of informed consent. Exclusion criteria included other forms of pulmonary hypertension (for example, postcapillary PH) or comorbidities (autoimmune diseases or interstitial lung disease with a well-defined association with precapillary PH) that could influence EV levels.

#### 2.2.2. Controls

Eighteen patients who had suffered PE and, after two years of follow-up, had not developed CTEPH were selected. Controls were matched with cases by age and sex. Inclusion and exclusion criteria mirrored those of the cases to ensure comparability.

The study was approved by the Ethics and Research Committee of the Virgen del Rocío Hospital in Seville, Spain (010470). All participants provided informed consent prior to enrollment.

#### 2.2.3. Variables

*Primary Variables:* Levels of EVs marked with CD90+, CD45+, and CD146+, quantified via flow cytometry.

*Secondary Variables*: Demographic characteristics (age, sex), body mass index, relevant medical history (hypertension, obstructive sleep apnea, bronchial asthma, cancer, psychiatric disorders), and whether the venous thromboembolism event was provoked.

*Data Collection*: Clinical data and blood samples were obtained from all participants. Blood samples were collected under standardized conditions to ensure consistency in EV analysis.

### 2.3. Platelet Poor Plasma Preparation

Venous blood was collected (discarding the first 3 mL) and placed in 3.5 mL of 9NC coagulation 3.2% sodium citrate Vacuette^®^ tubes (Greiner bio-one, Monroe, NC, USA). The tubes were maintained in a vertical position without agitation, and the blood samples were processed within 2 h of extraction. Platelet-poor plasma (PPP) samples were obtained by centrifugation as described previously [28,29,30]. Briefly, the samples were centrifuged at 1500× *g* for 30 min at 4 °C without brake. The collection of PPP (supernatant) was stopped 1 cm above the buffy coat to avoid cell contamination. The PPP samples were gently mixed, aliquoted, and stored at −80 °C for later EV quantification.

### 2.4. EVs Quantification

EV quantification was performed at the Institute of Biomedicine of Seville using a BD LSRII Fortessa flow cytometer (Becton Dickinson Biosciences, Erembodegen, Belgium). Briefly, 30 µL of frozen PPP were thawed at room temperature and incubated for 30 min at 20–25 °C with specific monoclonal antibodies: CD45-fluorescein isothiocyanate (FITC, Beckman Coulter, Marseille, France), CD146-FITC (AbD Serotec, Hercules, CA, USA), CD90-allophycocyanin (APC, Exbio, Vestec, Czech Republic), CD44-APC (Exbio, Vestec, Czech Republic), CD105-phycoerythrin (PE, Exbio, Vestec, Czech Republic). In parallel, samples were also incubated with their matched isotype and were used as negative controls. After that, 2 µL of Annexin-CF Blue (Inmunostep, Salamanca, Spain) was added to each tube and incubated for 15 min at room temperature with Annexin V binding buffer (Inmunostep, Salamanca, Spain). To limit background noise from dust and crystals, all reagents were filtered twice with 0.22 µm filters. Megamix-Plus SSC (Biocytex, Marseille, France), a mix of fluorescent polystyrene beads (0.16, 0.20, 0.24, and 0.5 μm), was used to calibrate the flow cytometer and to define an EV region, according to the manufacturer’s specifications. These beads are specifically designed to calibrate the flow cytometers for EV measurements. We considered EVs as events inside the EV region and positive for Annexin V. Endothelial-derived EVs as CD146+, leukocyte-derived as EV immune reactive for CD45+, CD44+, and EV compatible with mesenchymal origin as EV immune reactive for CD90+, CD105+. To quantify the absolute number of EVs, 30 μL of counting beads (6 μm) with a known concentration (approximately 1000 beads/μL; Perfect-Count Microspheres; Cytognos, Salamanca, Spain) were added to each sample. All samples were processed in the flow cytometer for 2 min at a low flow rate. Results are expressed as events/µL. Based on findings from the animal study, EVs labeled with CD146+, CD45+, and CD90+ were quantified in human samples.

### 2.5. Statistical Analysis

Categorical variables are presented as frequencies and percentages, while continuous variables are presented as medians with interquartile ranges (25th–75th percentile). EV results are expressed as events per microliter (events/μL). Statistical analyses were conducted using IBM SPSS version 26.0 and R Core Team (2023).

In the experimental study, hemodynamic changes were analyzed using generalized linear mixed models, treating the experimental model as a random factor. The Akaike Information Criterion (AIC) was used to select the best model for the variable of interest.

Assumptions of constant variance over time and normality of residuals were verified using normal probability plots (Q-Q plots) and the Shapiro–Wilk test. For the mean PAP variable at different follow-up times, a generalized linear mixed model with a gamma distribution and a logarithmic link function was applied using the ‘glmmTMB’ package in R. Spearman’s rho correlation coefficients were additionally calculated.

In the case-control study, the Mann–Whitney U test for independent samples was used to assess differences in the distribution of variables between groups. Additionally, multivariate analysis and logistic regression were conducted to identify EVs associated with disease status test hypotheses. A *p*-value of less than 0.05 was considered statistically significant.

## 3. Results

### 3.1. Experimental Study

**Figure 1:**
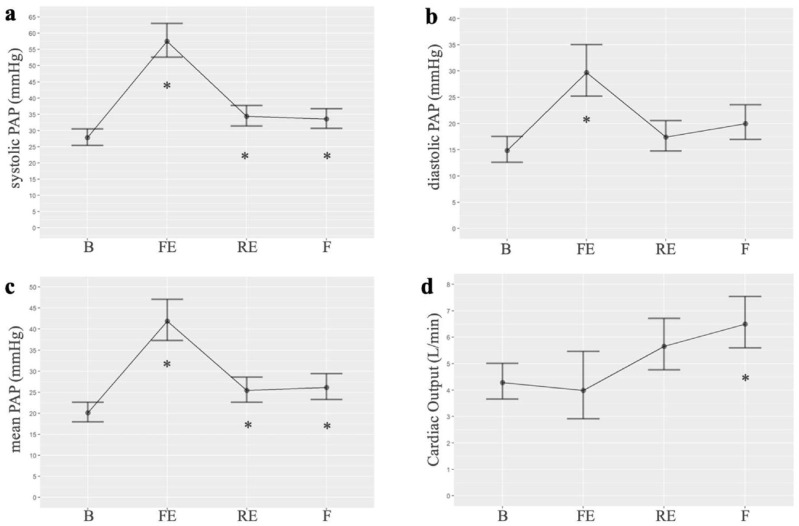
Normalized linear models of hemodynamic parameters measured at four points in the intervention group of the experimental study: (**a**). Systolic PAP (*n* = 7 for all timepoints); (**b**). Diastolic PAP (*n* = 7 for all timepoints); (**c**). Mean PAP (*n* = 7 for all timepoints); (**d**). Cardiac output (B *n* = 8, FE *n* = 2, RE *n* = 7, F *n* = 9). B: basal; FE: first embolization; RE: repeated embolization; F: final; PAP: pulmonary arterial pressure. *: *p* statistically significant compared to the basal measurement.

Among the hemodynamic changes produced after embolizations, we observed an increase in systolic, diastolic, and mean PAP compared to the baseline. The highest value of mean PAP was observed after the first embolization (mean 41.46 vs. 20.1 mmHg, *p* < 0.001). The mean PAP remained elevated, compared to baseline, after repeated embolizations (mean: 25.43, *p* = 0.02) and at the end of the experiment (mean: 26.14, *p* = 0.01). In the generalized linear mixed models, the best AIC was 175.4, 173.5, and 172.8 for systolic, diastolic, and mean PAP, respectively. The analysis of variance (ANOVA) for the mean PAP variable revealed significant differences across the three times studied. Additionally, cardiac output suffers a small decrease compared to baseline but increases progressively at the end of the experiment. Pulmonary hypertension was demonstrated in the pigs at the end of the experimental study (after several embolizations performed once a week). Figure 1 shows the results of the hemodynamic parameters. Table S1 presents the PAP values, while Table S2 compares the mean PAP at different times, as detailed in the Supplementary Materials.

Regarding EVs, we observed a consistent increase in their total quantification throughout the experiment. Notably, the highest EV quantification was recorded after the first embolization procedure, which aligned with the observed hemodynamic results. The acute increase in mean PAP observed after the first embolization (ΔmPAP + 22 mmHg) is consistent with the absence of vascular preconditioning and mimics an acute pulmonary embolism scenario. Progressive pulmonary hypertension developed with subsequent embolizations, as demonstrated by a significant correlation between embolization sequence and mPAP elevation (r = 0.76, *p* < 0.001), as well as an increase in right ventricular (RV) systolic pressure (from 42 ± 6 to 58 ± 8 mmHg), indicating RV adaptation over time.

**Figure 2:**
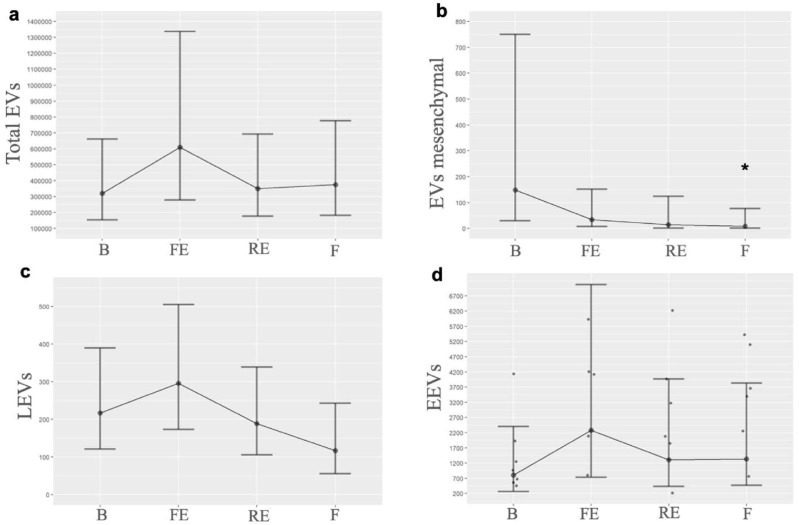
**Graphical representation of EV levels**. Behavior of EVs at the different times measured, highlighting their change in the first embolization and subsequently when pulmonary hypertension progressively develops: (**a**). Total EVs (B *n* = 6, FE *n* = 6, RE *n*= 7, F *n* = 6); (**b**)**.** EVs mesenchymal (B *n* = 7, FE *n* = 7, RE *n* = 6, F *n* = 5); (**c**)**.** LEVs (B *n* = 5, FE *n* = 6, RE *n* = 5, F *n* = 3); (**d**)**.** EEVs (B *n* = 8, FE *n* = 8, RE *n* = 8, F *n* = 9). B: basal; FE: first embolization; EEVs: endothelial extracellular vesicles; LEVs: leukocyte extracellular vesicles; EVs: extracellular vesicles; F: final; RE: repeated embolization. *: *p* statistically significant compared to the basal measurement.

**Figure 3:**
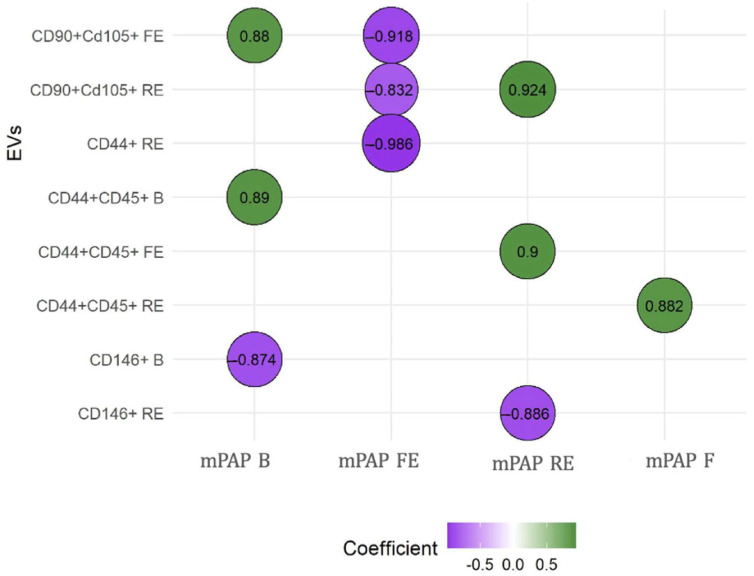
**Correlations between mean PAP and extracellular vesicles**. The relationship between EVs and mean PAP was measured using the Spearman rho test. We observed that CD146+ was the only one that showed negative correlations when PH was instigated. B: basal; EVs: extracellular vesicles; F: final; FE: first embolization; mPAP: mean pulmonary arterial pressure; RE: repeated embolization. CD90+ CD105+: EV compatible with mesenchymal origin; CD44+ CD45: leukocyte EV; CD146: endothelial EV. These analyses were performed to explore the potential predictive value of EVs in relation to the severity of mean pulmonary artery pressure (mPAP) measured at different timepoints.

When we quantified each EV by its cellular origin, we observed that EVs consistent with mesenchymal origin exhibited a progressive decrease from the first embolization, falling below baseline levels, with a *p*-value of 0.05 between the baseline and the final time. LEVs showed an initial increase after the first embolization but decreased once PH was established; moreover, by the end of the experiment, they fell below baseline. EEVs demonstrated a notable increase after the first embolization, and, although their levels decreased subsequently, they remained elevated compared to baseline. Table 1 shows the EVs’ quantification. Figure 2 illustrates the EV levels at the different times measured, and Figure 3 illustrates the behavior of mesenchymal-origin EVs and LEVs during hemodynamic changes.

EVs consistent with a mesenchymal origin showed a positive correlation with baseline and post-embolization mean PAP. LEVs stand out as the only ones that always show a positive correlation, suggesting their influence in the maintenance and perpetuation of PH. In contrast, EEVs demonstrated a negative correlation with mean PAP at baseline and after repeated embolization, once PH was established. The results of these correlations between the different EVs and mean PAP are presented in Figure 3. Supplementary Table S3 shows all correlations between EV subtypes and mean PAP. RV remodeling was clearly evident in embolized animals, and histological analysis further confirmed disease features, with organized thrombi detected in 78% of embolized pulmonary arteries and marked muscularization of distal pulmonary arterioles (67 ± 12% vs. 22 ± 8% in controls); these findings are shown in Supplementary Figure S1. Our working group has published these results previously [31].

### 3.2. Clinical Case-Control Study

Clinical characteristics of CTEPH patients were a mean age of 55.6 (±20.2) years, females 55%, and one patient (11.1%) had a history of cancer. Of them, four patients underwent thromboendarterectomy surgery; however, residual thrombosis was present in two of them without residual PH. EVs were quantified after surgery. Controls’ clinical characteristics were a mean age of 58 (±18) years, a predominance of females (55%), and six (33.3%) patients had a history of cancer. Demographics and baseline clinical characteristics of patients are provided in Table 2.

**Figure 4:**
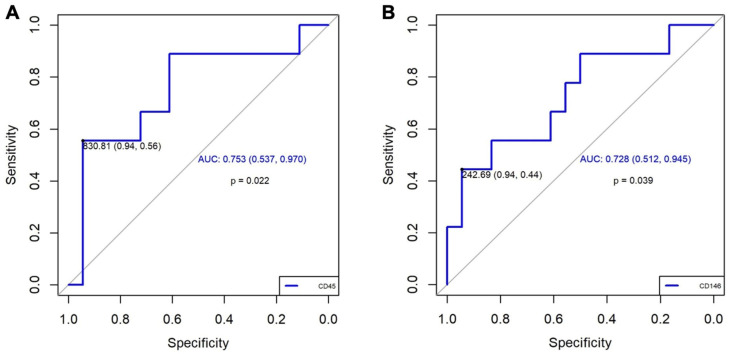
**ROC curve results**. (**A**): For CD45+, we selected a cut-off point based on a value of 830.81, which gives us a high sensitivity (0.94) and a moderate specificity (0.56). The AUC of 0.753 (95% CI: 0.537–0.970) suggests a moderate general discrimination capacity. (**B**): For CD146+, we selected a cut-off point based on a value of 242.69, which gives us a high sensitivity (0.94) and a moderate specificity (0.44). The AUC of 0.728 (95% IC: 0.512–0.945) also suggests a moderate general discrimination capacity. AUC: area under the curve; IC: confidence interval; ROC: Receiver Operating Characteristic.

Based on the results obtained in the experimental study, we performed the quantification of EVs marked with CD90+, CD45+, and CD146+. Concerning the quantification of EVs in patients, we observed a significantly higher increase in CD45+ (*p* = 0.035) and a trend towards higher increase in CD146+ (*p* = 0.059) in patients with CTEPH compared with patients who had only presented PE. These results were not modified by the associated comorbidities in either group. (Table 2). The value of EV CD90+ was very low, and it was not possible to compare with the other two EVs. Additionally, the diagnostic accuracy of CD45+ and CD146+ for CTEPH generation was good based on the area under an ROC curve (0.753, 95% CI: 0.54–0.96; and 0.728, 95% CI: 0.51–0.93; respectively, Figure 4). A value of CD45+ ≥ 830.81 demonstrated an OR of 21.25 (95% CI: 1.91–236.00; *p =* 0.013), a sensitivity of 56%, a specificity of 94%, a positive predictive value (PPV) of 83.3%, and a negative predictive value (NPV) of 81%. Meanwhile, a value of CD146+ ≥ 242.7 demonstrated an OR of 13.60 (95% CI: 1.22–151.04; *p =* 0.034), a sensitivity of 44%, a specificity of 94%, a PPV of 80%, and an NPV of 77%, Table 3. These results suggest that CD45+ has a better association with CTEPH than CD146+.

## 4. Discussion

Our experimental design effectively induced a model of chronic post-embolic pulmonary hypertension, as evidenced by significant alterations in hemodynamic parameters, including systolic, diastolic, and mean pulmonary arterial pressures. Notably, the utilization of large animal models for PH research is relatively uncommon, enhancing the translational relevance of our findings [32,33].

The results from the experimental model showed that mesenchymal origin EVs and LEVs decrease and fall below the basal level when PH is induced, while EEVs exhibit a different expression, maintaining persistently elevated levels. We observed that the EEVs in the clinical case-control study were elevated in CTEPH patients compared to the control group.

EEVs present a tissue factor (TF) on the surface, a powerful initiator of procoagulation and thrombogenic activity, increasing the risk of venous thrombosis. EEVs might be released from various sources, including interleukin-6 (IL-6) or tumor necrosis factor α (TNF-α), with the latter causing proteolytic activity associated with plasmin that favors cell migration and adhesion. It has also been shown that sphingosine 1-phosphate (S1P) strongly enhances the expression of TF, and, in turn, S1P is involved in the process of angiogenesis and inflammation [34,35]. Amabile et al. [36] demonstrated that patients with precapillary PH present an increase in the number of various populations of circulating EEVs and LEVs; therefore, the presence of a greater number of EEVs may be related to the structural damage of the endothelium in PH, as we observed in our results.

Lv et al. [21] have described an increase in TNF-α, NF/kB, and the anti-apoptotic protein BCL2. Furthermore, it is widely accepted that inflammation is a powerful pathological driver of susceptibility and progression of vascular remodeling in PAH, as well as all severe forms of PH. Therefore, inflammation likely progresses as an adaptive or maladaptive response to attempt to restore homeostasis, affecting neovascularization and producing angiogenesis [37].

On the other hand, inflammation has a determining role in the generation of PH through cytokines such as IL-6 and growth factors. These activate the Janus Kinase (JAK) pathway and the STAT, which are involved in the remodeling of pulmonary arteries through differentiation, proliferation, and apoptosis [38]. Karpov et al. [39] inhibited JAK 1/2 in a murine model; however, there are also other experimental models that use ruxolitinib (JAK/STAT inhibitor) or other signaling blockers by reducing the P-STAT3, P-PI3K, and c-Myc pathways [40]. In this sense, recently, an inhaled tyrosine kinase inhibitor, seralutinib, has just passed a phase 2 clinical trial to treat PAH [41,42].

Various studies establish that CD44+, with the activity of PI3 through Rho-kinase (ROK), achieves adhesion and migration of endothelial cells as well as angiogenesis, inducing PI3K [43]. It seems that CD45+ can influence the expression of CD44+ [44]. Mononuclear cells recruited to the pulmonary vessel wall contribute to the changes in fibroblast proliferation status observed in response to chronic hypoxic exposure through the release of numerous growth factors, in addition to a marked production and accumulation of the extracellular matrix, especially type I collagen, in hypoxia-induced vascular remodeling [44].

LEVs stimulate the expression of proinflammatory genes in endothelial cells, leading to the production of cytokines and leukocyte adhesion molecules to endothelial cells in vitro. Both LEVs and EEVs positively regulate the production of proinflammatory mediators, generate endothelial dysfunction due to an increase in reactive oxygen species [45], and negatively regulate the PI3K and NFκB pathway [46]. Gąsecka et al. have demonstrated how prostacyclin analogues cause a decrease in the release of LEVs and platelet origin, causing a greater risk of bleeding due to the alteration in platelet function [47].

Although MSCs have a wide range of markers, Firth et al. [48] have studied endarterectomized tissue from patients with CTEPH, according to the criteria of the International Society of Cellular Therapy [49]. They determined positive cell surface markers that included CD105 and CD90 with the capacity to generate multiple lineages. Myofibroblasts were observed (expressing α-smooth muscle actin or α-SMA), different from smooth muscle precursor cells, but that activate inflammatory cytokines and proangiogenic factors, which have powerful effects on MSCs of vascular tissue. Although there are possibly factors external to the unresolved clot that could be a stimulus on their own to trigger said cellular deregulation and the development of CTEPH, we observed in our experimental model that EVs related to a mesenchymal origin, CD90+, present an increase and maintains it until the end, which may suggest greater activity of the mesenchymal cells that intervene in apoptosis to maintain CTEPH, while CD105+ EVs presents a progressive decrease.

We emphasize the strength of our study: it is the first to compare data from an experimental model and extrapolate it to routine clinical practice. Our study has some limitations. First, the definition of EV levels is not standardized, and we believe it is essential to generate a robust scientific basis to enhance the interpretation of the results. Furthermore, our study was conducted prior to the publication of the 2018 MISEV guidelines, which now define best practices in EV research; however, our protocol focused on phosphatidylserine-positive EVs (Annexin V+) as a validated surrogate at the time. Second, due to all the information that EVs can contain, it might be necessary to obtain more detailed results on the specific mechanisms that influence vascular remodeling. Third, our study does not quantify inflammatory markers; therefore, future studies should incorporate inflammatory markers along with EV quantification to establish this link more definitively. Finally, case-control study has the limitations inherent to their design; undoubtedly, to quantify EVs during the follow-up of PE and the development of CTEPH through prospective studies would be interesting

## 5. Conclusions

Our translational study proposes that LEVs and EEVs play a crucial role in sustaining the vascular alterations leading to pulmonary vascular remodeling in CTEPH. These results open new avenues for research, suggesting that intercellular communication facilitated by EVs could be pivotal in the progression of CTEPH and the development of future therapeutic strategies.

## Figures and Tables

**Table 1:** Results of EVs’ quantification in the experimental study.

EVs/Time	B	FE	RE	F
Total EVs	319,748.0 (118,608.0)	610,245.7 (244,310.6)	350,841.1 (121,874.2)	375,911.3 (139,032.1)
Consistent with mesenchymal-origin, *mean* (*SE*)	149.33 (25.3)	34.28 (4.9)	15.14 (4.4)	8.34 (2.68)
LEVs, *mean* (*SE*)	217.30 (4.8)	295.81 (5.5)	189.17 (4.2)	116.88 (4.09)
EEVs, *mean* (*SE*)	802.12 (62.9)	2274.57 (190.7)	1307.6 (104.8)	1335.11 (96.8)

B: basal; EVs: extracellular vesicles; EEVs: endothelial extracellular vesicles; FE: first embolization; F: final; LEVs: leukocyte extracellular vesicles; RE: repeated embolizations; SE: standard error.

**Table 2:** Baseline characteristics of patients in the case-control study and results of EVs quantification.

Variables	Cases (n = 9)	Controls (n = 18)
Age, mean (SD)	55.6 (±20.2)	58 (±18)
Sex female, n (%)	5 (55.6)	10 (55.6)
BMI, mean (SD)	29 (±6.1)	30.5 (±5.3)
Arterial hypertension, n (%)	1 (11.1)	1 (5.5)
OSA, n (%)	2 (22.2)	0
Bronchial asthma, n (%)	1 (11.1)	0
Cancer, n (%)	1 (11.1)	6 (33.3)
Psychiatric disease, n (%)	0	2 (11)
VTE provoked, n (%)	5 (55.5)	12 (66.7)
**Quantification of Extracellular Vesicles**		
CD45+ (P25-75)	875,1986 (504,8326–1033,7861)	474,7100 (378,7331–639,4884) *
CD146+ (P25-75)	198,1521 (90,9730–1317,2157)	85,8275 (50,2293–159,3167) *

Baseline characteristics of patients in the case-control study and results of EVs quantification. BMI: body mass index, OSA: obstructive sleep apnea; VTE: venous thromboembolism. * *p* value < 0.05.

**Table 3:** The results of the diagnostic characteristics of EVs in the case-control study.

	CD45+ (95% CI)	CD146+ (95% CI)
Odds ratio	21.25 (1.91–236.00)	13.60 (1.22–151.04)
Sensitivity, %	94 (84–105)	94 (84–105)
Specificity, %	56 (23–88)	44 (12–77)
Positive predictive value, %	83 (53–103)	80 (45–115)
Negative predictive value, %	81 (64–98)	77 (60–95)
Positive probability ratio, %	10.00	10.00
Negative probability ratio, %	0.47	0.47

**Logistic regression results**. CD45+ demonstrated an OR of 21.25 (95% CI: 1.91–236.00; *p* = 0.013), sensitivity of 94%, and specificity of 56%, with a high positive and negative predictive value (83.3% and 81%, respectively). CD146+ demonstrated an OR of 13.60 (95% CI: 1.22–151.04; *p* = 0.034), sensitivity of 44%, and specificity of 94%, with a high positive and negative predictive value (80% and 77%, respectively).

## Data Availability

The data presented in this study are available upon request from the corresponding author due to ethical reasons.

## Supplementary Materials

The following supporting information can be downloaded at https://www.mdpi.com/article/10.3390/biomedicines13061499/s1, Table S1: Summary of all measured PAP values; Table S2: Comparisons between mean PAP at different times; Table S3: Correlation between extracellular vesicle subtypes and mean PAP; Figure S1: Histological changes in the pulmonary arteries of pigs subjected to the experimental model of chronic post-embolic pulmonary hypertension.

## Abbreviations

The following abbreviations are used in this manuscript:

α-SMA	α-smooth muscle actin
AIC	Akaike Information Criterion
ANOVA	Analysis of variance
B	Baseline
CCL2	Chemotactic protein-1
CTEPH	Chronic thromboembolic pulmonary hypertension
EEVs	Extracellular vesicles of endothelial origin
EVs	Extracellular vesicles
F	Final
FE	First embolization
IL-6	Interleukin-6
JAF	Janus Kinase
LEVs	Extracellular vesicles of leukocyte-derived
miRNA	microRNA
MSCs	Mesenchymal stem cells
NF-κB	Nuclear factor-κB
NLRP3	Nucleotide-binding oligomerization domain-like receptor protein 3
NPV	Negative predictive value
PAP	Pulmonary arterial pressure
PE	Pulmonary embolism
PH	Pulmonary hypertension
PI3K	Phosphatidylinositol 3-kinase
PPV	Positive predictive value
Q-Q plots	Normal probability plots
RV	Right ventricular
S1P	Sphingosine 1-phosphate
STAT	Signal transducer and activator of transcription
TF	Tissue factor
TNF-α	Tumor necrosis factor-α
VEGF	Vascular endothelial growth factor
