# Supplementary material for: Role of Extracellular Vesicles in Chronic Post-Embolic Pulmonary Hypertension: Data from an Experimental Animal Model and Patients
Source: Biomedicines. 2025 Jun 18;13(6):1499. doi: 10.3390/biomedicines13061499 (PMC12191311; doi:10.3390/biomedicines13061499)
Supplement: Supplementary file 1 [file biomedicines-13-01499-s001.zip › biomedicines-3668014-supplementary.pdf]

Table S1. Summary of all measured PAP values.

Time	Mean ± SE (95% IC)
**Systolic PAP**	
Basal	27.85 ± 1.28 (25.44 – 30.49)
First embolization	57.56 ± 2.66 (52.58 – 63.02)
Repeated embolization	34.41 ± 1.59 (31.43 – 37.68)
Final	33.53 ± 1.55 (30.62 – 36.72)
**Diastolic PAP**	
Basal	14.86 ± 1.25 (12.60 – 17.51)
First embolization	29.71 ± 2.49 (25.20 – 35.03)
Repeated embolization	17.43 ± 1.46 (14.78 – 20.54)
Final	20.0 ± 1.67 (16.96 – 23.58)
**Mean PAP**	
Basal	20.14 ± 1.20 (17.92 – 22.63)
First embolization	41.86 ± 2.49 (37.24 – 47.03)
Repeated embolization	25.42 ± 1.51 (22.63 – 28.57)
Final	26.14 ± 1.55 (23.26 – 29.38)

Results of the generalized linear mixed model: mean value of the different pressures at the four measured times. PAP: pulmonary arterial pressure; SE: standard error.

Table S2. Comparisons between mean PAP at different times.

Comparisons	ratio ± SE	z-ratio
Basal and first embolization	0.48 ± 004	-8.69 *
Basal and repeated embolization	0.79 ± 0.06	-2.77 *
Basal and final	0.77 ± 0.06	-3.09 *
First and repeated embolization	1.64 ± 0.13	5.92 *
First embolization and final	1.60 ± 0.13	5.59 *
Repeated embolization and final	0.97 ± 0.08	-0.33

Results of the generalized linear mixed model: We performed multiple comparisons between the mean PAP at different times measured. This table shows the ratio and its respective SE in addition to the z-ratio. SE: standard error. *: *p* statistically significant.

Table S3. Correlation between extracellular vesicle subtypes and mean PAP.

EV	mPAP Basal	mPAP FE	mPAP RE	mPAP Final
Total EV basal	0.093	-0.464	0.288	0.174
Total EV FE	-0.154	**-0.812***	0.638	0.203
Total EV RE	0.370	-0.319	0.522	-0.087
Total EV Final	0.000	-0.872	**0.975***	0.667
CD90+ CD105+ basal	-0.206	-0.432	0.667	0.180
CD90+ CD105+ FE	**0.880***	**-0.918***	0.487	0.072
CD90+ CD105+ RE	0.197	**-0.832***	**0.924***	0.554
CD90+ CD105+ Final	0.406	0.289	0.632	0.263
CD44+ basal	0.337	-0.396	0.180	-0.198
CD44+ FE	0.299	-0.126	0.450	0.432
CD44+ RE	-0.463	**-0.986***	0.754	0.754
CD44+ Final	0.474	0.154	-0.154	0.103
CD44+ CD45+ basal	**0.894***	-0.462	0.700	0.100
CD44+ CD45+ FE	0.759	-0.493	**0.886***	-0.143
CD44+ CD45+ RE	0.783	-0.616	**0.900***	**0.882***
CD44+ CD45+ Final	-0.866	0.866	-0.780	-0.500
CD146+ basal	**-0.874***	-0.270	-0.036	0.631

CD146+ FE	-0.655	-0.270	0.036	0.613
CD146+ RE	-0.088	0.657	**-0.886***	-0.580
CD146+ Final	-0.337	-0.126	-0.090	0-162

This table summarizes all Spearman correlation coefficients between EV counts (by subtype and timepoint) and mPAP measurements at baseline, after first embolization (FE), repeated embolizations (RE), and at the final timepoint. Both time-matched and cross-timepoint associations are included to explore potential predictive relationships.

Abbreviations: EVs: extracellular vesicles; FE: first embolization; mPAP: mean pulmonary arterial pressure; RE: repeated embolization. CD90+ CD105+: EV compatible with mesenchymal origin; CD44+ leukocyte-derived EV; CD44+ CD45+: leukocyte-derived EV; CD146: endothelial EV. **p* statistically significant.

Figure S1. Histological changes in the pulmonary arteries of pigs subjected to the experimental model of chronic post-embolic pulmonary hypertension.

a) b)

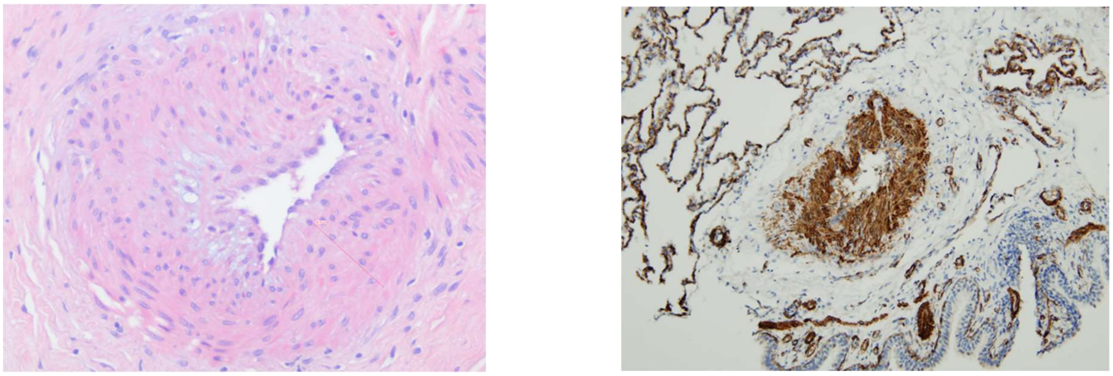

Histological samples of the pulmonary arteries in the experimental model, a) hematoxylin-eosin staining; b) SMA-smooth muscle actin staining. These histological samples correspond to distal pulmonary arteries, from the experimental study, after the completion of embolization to verify vascular remodeling when pulmonary hypertension was established.
